# Association Between Mitochondrial Function and Rehabilitation of Parkinson's Disease: Revealed by Exosomal mRNA and lncRNA Expression Profiles

**DOI:** 10.3389/fnagi.2022.909622

**Published:** 2022-06-16

**Authors:** Yixuan Wang, Yonghong Liu, Zhaohui Jin, Cui Liu, Xin Yu, Keke Chen, Detao Meng, Aixian Liu, Boyan Fang

**Affiliations:** ^1^Parkinson Medical Center, Beijing Rehabilitation Hospital, Capital Medical University, Beijing, China; ^2^Beijing Rehabilitation Medical College, Capital Medical University, Beijing, China

**Keywords:** Parkinson's disease, rehabilitation, exosome, messenger RNA, NDUFB7, mitochondrial function, long noncoding RNAs

## Abstract

Rehabilitation has been proposed as a valid measure complementary to the management of Parkinson's disease (PD). However, the mechanism underlying is not clear yet. The differential expressions of exosomal messenger RNA (mRNA) and long noncoding RNAs (lncRNAs) may play a critical role in PD progression and rehabilitation. To compare the differential expressions of exosomal mRNAs and lncRNAs, patients with PD (PWPs, Hoehn and Yahr stages 1.5-2.5, *n* = 6) and age- and sex-matched healthy controls (HCs, *n* = 6) were included in this study. All PWPs received a 2-week rehabilitation treatment in the hospital, which seemingly led to improvement in both the motor and non-motor functions. A set of differentially expressed mRNAs (DEmRNAs) and differentially expressed lncRNAs (DElncRNAs) extracted from exosomes in blood samples *via* next-generation sequencing (NGS) was screened out. Compared to HCs, 2,337 vs. 701 mRNAs and 1,278 vs. 445 lncRNAs were significantly upregulated and significantly downregulated, respectively, in pre-rehabilitation (pre-rehab) PWPs; 2,490 vs. 629 mRNAs and 1,561 vs. 370 lncRNAs were significantly upregulated and significantly downregulated, respectively, in post-rehabilitation (post-rehab) PWPs. Compared to pre-rehab PWPs, 606 vs. 1,056 mRNAs and 593 vs. 1,136 lncRNAs were significantly upregulated and significantly downregulated, respectively, in post-rehab PWPs. Overall, 14 differentially expressed mRNAs (DEmRNAs) and 73 differentially expressed lncRNAs (DElncRNAs) were expressed in the blood exosomes of HCs, pre- and post-rehab PWPs, simultaneously. Gene Ontology (GO) and Kyoto Encyclopedia of Genes and Genomes (KEGG) pathway enrichment analyses identified 243 significantly co-expressed lncRNA-mRNA pairs. One DEmRNA of interest (ENSG00000099795, NDUFB7) and three corresponding DElncRNAs (ENST00000564683, ENST00000570408, and ENST00000628340) were positively related. Quantitative real-time polymerase chain reaction (qRT-PCR) validated that the expression levels of NDUFB7 mRNA and the 3 DElncRNAs increased significantly in pre-rehab PWPs, but decreased significantly in post-rehab PWPs compared to HCs. NDUFB7 mRNA is a marker related to mitochondrial respiration. It is reasonably believed that mitochondrial function is associated with PD rehabilitation, and the mitochondrial pathway may involve in the pathogenesis of PD.

## Introduction

Parkinson's Disease (PD) is one of the fastest-growing neurological disorders characterized by bradykinesia, rigidity, and tremor (Bloem et al., 2021). The primary pathological changes underlying PD are the degeneration of dopaminergic neurons in the substantia nigra and significant decreases of dopamine in the striatum (Ntetsika and Papathoma, 2021). The neurodegenerative pathogenesis may be related to oxidative stress (Puspita and Chung, 2017), genetic predisposition (Pinnell et al., 2021), apoptosis (Venderova and Park, 2012), and neuroinflammation (Tansey and Goldberg, 2010). Increasing evidence has indicated that rehabilitation might be a promising supplementary therapy to prevent PD development and delay PD progression (Xu et al., 2019). However, the potential mechanisms of PD, especially the mechanism underlying PD rehabilitation, are not clear yet. High-quality, objective biomarkers are needed for accurate PD evaluation.

Exosomes (EXOs) are intracellular membrane-based vesicles with diverse compositions involved in biological and pathological processes (Doyle and Wang, 2019), including intercellular communication, the regulation of cellular function, and waste management (Van Der Pol et al., 2012). Various important cell signaling molecules, such as messenger RNA (mRNA), long non-coding RNAs (lncRNA), and proteins can be transported and transmitted by EXOs (Cheng et al., 2021). The contents of exosomes are stable and can be transported into receiver cells to exert their biological roles (Cheng et al., 2021). Therefore, exosomes are considered potential biomarkers for diagnosing various diseases, such as PD, e.g., miR-133b variant, a marker involved in dopamine neuron survival in mice (Kim et al., 2007; Lee et al., 2019). A previous study suggested that lnc-MKRN2-42:1 may be involved in the occurrence and development of PD (Wang et al., 2020). Considering the properties of exosomes, it is appropriate to use RNA-based biomarkers from EXOs to study PD.

Mitochondrial dysfunction has been observed in PD (Macdonald et al., 2018). The nigral neurons are highly susceptible to mitochondrial dysfunction due to high basal rates of oxidative phosphorylation leading to increased oxidative stress (Haddad and Nakamura, 2015). Mitochondrial dysfunction, especially reductions in complex I activity has been evidenced in both familial and sporadic forms of PD (Haddad and Nakamura, 2015). Complex I activity may become an essential marker of the development and assessment of PD. However, the expression of mRNAs and lncRNAs, and their interactions underlying mitochondrial mechanisms in peripheral blood exosomes of PWPs before and after rehabilitation remains unknown.

In this study, we aimed to compare the expression profiles of exosomal lncRNA and mRNA in the peripheral blood between healthy controls (HCs), pre-and post-rehab PWPs for the purpose to elucidate their potential association with PD pathogenesis and rehabilitation. The differentially expressed mRNAs (DEmRNAs) and differentially expressed lncRNAs (DElncRNAs) of interest were characterized to identify their potential function and signaling pathways in PD rehabilitation.

## Materials and Methods

### Subjects and Sample Collection

We included 80 PWPs between June 2020 and December 2020 in the Parkinson Medical Center of Beijing rehabilitation hospital. Six PWPs who met the inclusion criteria and were willing to participate in the project were included. Six age- and sex-matched healthy subjects were also included as HCs. Most HCs were life partners or caregivers of the PWPs.

All PWPs met the clinical diagnosis of PD based on the Movement Disorder Society (MDS) diagnostic criteria for primary PD (Postuma et al., 2015), with a disease severity rating of stage 1.5 to 2.5 on the Hoehn and Yahr (H&Y) scale (1 to 5 points from mild to severe) during the “ON” stage (Hoehn and Yahr, 1967); <75 years of age; without comorbidities requiring treatment; without deep brain stimulation therapy; Mini-Mental State Exam (MMSE) score >24 if education level ≥ secondary school or >20 if education level ≤ primary school (Folstein et al., 1975); stable medication use; and the ability to walk and stand without assistance.

The PWPs were excluded if they had a history of other neurological, psychological, or medical conditions, including atypical PD; history of head injury (stroke, depression, or learning disability); debilitating conditions other than PD (e.g., hearing or vision impairment); or significantly abnormal laboratory tests (including hematology, electrolytes, renal and liver function tests).

**Figure 1:**
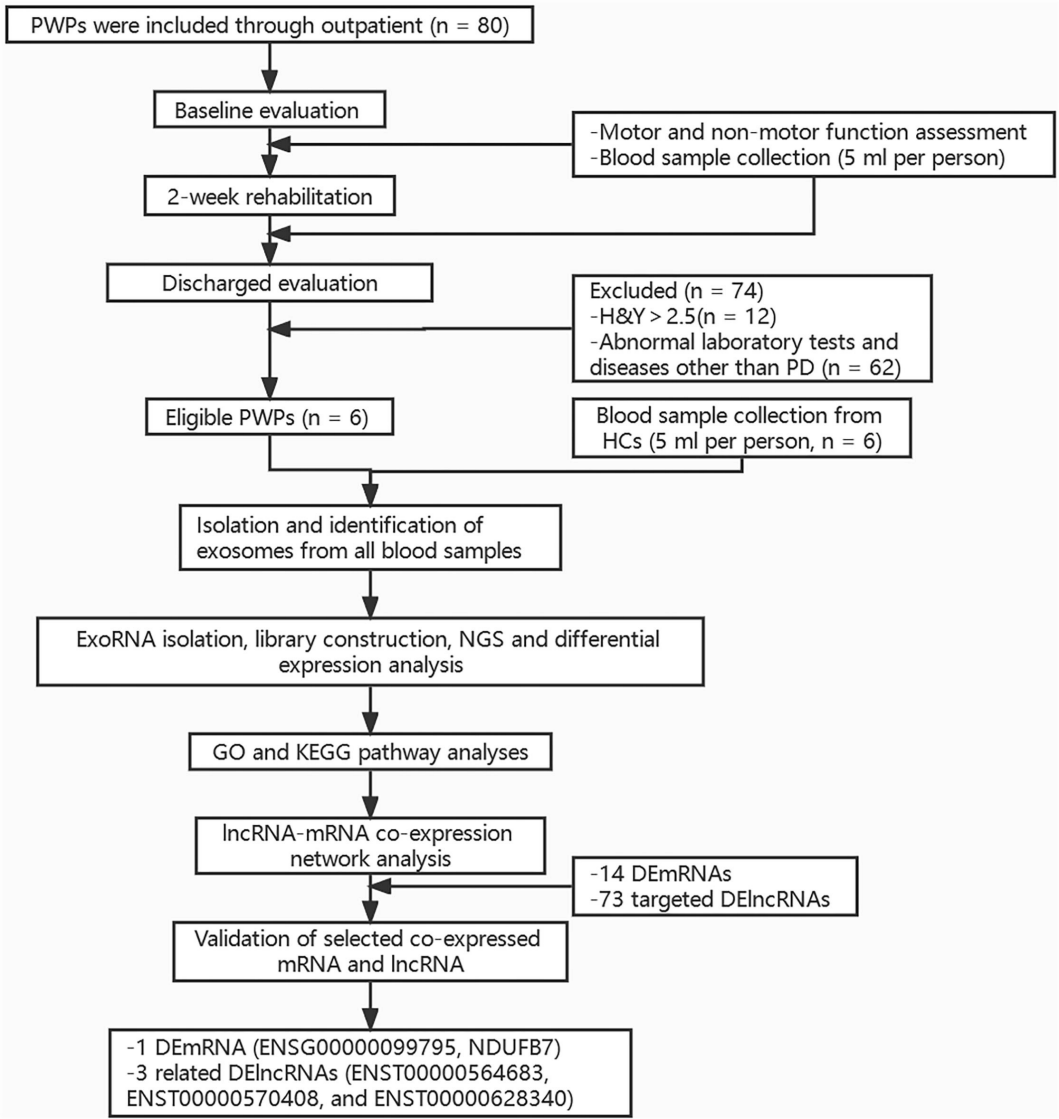
Flow chart of the study. PWPs, patients with Parkinson's disease; H and Y, Hoehn and Yahr; PD, Parkinson's disease; HCs, healthy controls; NGS, Next-generation sequencing; GO, Gene ontology Ontology; KEGG, Kyoto Encyclopedia of Genes and Genomes; DEmRNAs, differentially expressed mRNAs; DElncRNAs, differentially expressed lncRNAs.

Peripheral blood sample (5 ml) was taken from each participant under fasting conditions between 6 am and 7 am on Day 2 of hospitalization. All samples were stored at −80°C for later assay. The study protocol was approved by the Ethics Committee of Beijing Rehabilitation Hospital, Capital Medical University (approval No. 2020bkky010). All participants voluntarily signed informed consent. Flow chart of the study is shown in Figure 1.

### Rehabilitation Procedure

Rehabilitation was administered as previously described (Chen K. K. et al., 2021). Briefly, three to four patients with PD were admitted to hospital simultaneously for a two weeks rehabilitation covering four daily sessions, five days a week in hospital. The four sessions consisted of (1) one on one physical therapy; (2) balance and gait training using C-MiLL (Motekforce link, Culemburg, Netherlands) and Balance Tutor (Meditouch, Netanya, Israel); (3) aerobic training using upper and lower limb trainer (T5XR; Nustep, Ann Arbor, MI, USA); (4) speech therapy. Each session lasted for 30 to 60 min and run by one well-trained physical (for session1, 2, and 3) or speech therapist (for session 4) respectively to keep the consistency.

The total intervention time is 2.5 h per day. All sessions were divided and completed in the morning and afternoon (1–1.5 h for half a day). For example, the therapists conducted the C-Mill treadmill training (30 min) and one-on-one PT session (30 min) in the morning and conducted the balance tutor training (30 min), aerobic training (30 min), and speech therapy training (30 min) in the afternoon. The interval between sessions was at least 30–60 min, and the patients can get enough rest for the upcoming training. In addition, they will be asked if any discomfort appears on the next day such as muscle soreness. No feelings of fatigue were reported. Especially, we screened for cardiovascular endurance by cardiopulmonary exercise test for each patient before intervention to avoid risk. The whole study process lasted for about 1 year. The rehabilitation procedure for patients with PD is shown in Table 1.

**Table 1:** Illustration of rehabilitation procedure for patients with PD.

**Intervention**	**Description and dose**	**Selection and adjustment of intervention**
One on one physical therapy	This session was conducted by one well-trained therapist for 30 minutes per day: 1. warm-up activities (5 minutes): Stretching all the joints and major muscle 2. Active and passive exercises (20 min): 2.1 Stretching: extremities and spine stretching, ROM traction 2.2 Strength training: isometric training, isotonic training, resistance training 2.3 Balance training: tandem, one leg stance, inclined ramp 2.4 Gait training: external cueing (visual or auditory cues), dual-task training 2.5 Adjustment /control of posture: antigravity trunk extension 3 Cool-down (5 min): Stretching all the joints	• H and Y stage 1-2: Items 2.2, 2.3, and 2.4 are required. Difficulty was raised according to patients' adaptation to the intervention. For example, balance training performed on different surfaces like foam and inclined ramp and less cues were provided during gait training. • H and Y stage>2:All items are required. Difficulty was reduced according to the severity of symptoms.
Balance and gait training	The training was conducted by one well-trained therapist using automatized and standardized program supported by C-Mill (conducted in the morning for 30 min per day) and Balance Tutor (conducted in the afternoon for 30 min per day).	Difficulty was adjusted according to the severity of symptoms. For example, more reaction times or cues were provided for patients with worse function of gait or balance.
Aerobic training	The training was conducted by one well-trained therapist using upper and lower limb trainer (T5XR; Nustep, Ann Arbor, MI, USA) for 30 min per day.	Difficulty was adjusted according to patients' adaptation to the intervention. For example, increased resistance level was set for patients with good adaptation to the training.
Speech therapy	Three possible kinds of interventions were conducted by one speech therapist for 30 min per day: (1) counseling for the management of swallowing and language problems; (2) individual swallowing training (3) speech therapy to treat hypokinetic dysarthria	The determination of speech therapies for patients with PD was mainly based on their complaints and symptoms, as well as the results of swallowing angiography: • For patients with PD with mild dysphagia or dysarthria, only one kind of intervention was needed. • For patients with PD with both dysphagia and dysarthria, we gave two kinds of intervention (2 and 3).
Home exercise program	A platform of home exercise program for patients with PD was established by Beijing Rehabilitation Hospital, which consisted of several home exercise video courses.	patients with PD keep doing exercise at home and sign up every day after 2-week rehabilitation.

*PD, Parkinson's disease; ROM, range of motion; H and Y, Hoehn and Yahr*.

### Clinical and Neuropsychological Assessment

Demographic data were collected, including gender, age, and disease duration. All PWPs underwent a battery of assessments under “ON” medication state (1 h after taking their regular antiparkinsonian medication and having a good therapeutic effect) at baseline (pre-rehabilitation) and on Day 1 after a 14-day rehabilitation (post-rehabilitation). The assessments included motor function, cognitive function, quality of life, and neuropsychological symptoms necessary for the evaluation of PD motor and non-motor functions. The Movement Disorders Society–Unified Parkinson's Disease Rating Scale Part III (MDS-UPDRS-III; Chung et al., 2020), the 10-m walk test (10MWT; Tao et al., 2021), the 6-min walk test (6MWT; Koyanagi et al., 2021), the Five Times Sit-to-Stand Test (FTSST; Duncan et al., 2011), the Timed Up-and-Go Test (TUG; Giardini et al., 2018), and the Mini Balance Evaluation System Test (Mini-BESTest; Conradsson et al., 2015) were used to assess walking and balance function. The Mini-mental State Examination (MMSE; Zhao et al., 2020) and the Montreal Cognitive Assessment (MoCA; Van Steenoven et al., 2014) were used to assess cognitive function. The Hamilton Anxiety Scale (HAMA; Sumec et al., 2017) and the Hamilton depression scale (HAMD; Sumec et al., 2017) were used to evaluate psychiatric symptoms. The Parkinson's Disease Questionnaire (PDQ-39; Meng et al., 2022) was used to measure perceived health in terms of physical, mental, and social functions. The evaluations were performed randomly and patients were given short breaks between assessments.

### ExoRNA Isolation, Library Construction, and Next-Generation Sequencing (NGS)

ExoRNA isolation, library construction, and high-throughput sequencing were carried out by CloudSeq Biotech Inc. (Shanghai, China). Briefly, total RNAs were extracted, followed by the removal of ribosomal RNAs (rRNAs) using TRIzol reagent (Invitrogen, Carlsbad, CA, USA) and NEBNext® rRNA Depletion Kit (New England Biolabs, Inc., Massachusetts, USA) according to the manufacturer's instructions. The TruSeq Stranded Total RNA Library Prep Kit (Illumina, USA) was used for the construction of RNA libraries. The RNA libraries underwent quantitative and quantitative evaluation on the Agilent 2100 Analyzer (Agilent Technologies, USA). Ten pM libraries were denatured to single-stranded DNA molecules and were then captured on Illumina flow cells, amplified *in situ* as clusters, and sequenced with 150 cycles on Illumina NovaSeq 6000 sequencer following the manufacturer's protocol.

### Differential Expression Analysis

The raw reads were generated by Illumina NovaSeq 6000 sequencer. Low-quality reads were removed using fastp software (v0.20.0). STAR software (v2.7.9a) was used to align the high-quality clean reads to the human reference genome (GRCh38/hg38). Featurecount software (v2.0.2) and HTSeq software (v0.13.5) were used to obtain the raw read counts of mRNA gene level and lncRNA transcript level as the mRNA and lncRNA expression profiles, respectively. The fold change and *P*-value between two groups of samples were calculated using the DESeq2 R package, and the fold change ≥2.0 and a *p* ≤ 0.05 were categorized as the threshold for DEmRNA and DElncRNA screening.

### Gene Ontology (GO) and Kyoto Encyclopedia of Genes and Genomes (KEGG) Pathway Analyses

The GO and KEGG analyses were carried out for the DEmRNAs. GO analysis provides a controlled vocabulary for annotating and inferring the functions of DEmRNAs. The GO terms covered the common terms of biological process (BP), molecular function (MF), and cellular component (CC). The KEGG pathway analysis was used to speculate on the biological functions involved in DEmRNAs. In brief, the Ensembl human GTF gene annotation database (v104) was used to annotate the DEmRNAs. GO and KEGG pathway enrichment analyses were performed with clusterProfiler R package (v3.18.1) based on the DEmRNAs. A *p* < 0.05 was considered statistically significant.

### lncRNA–mRNA Co-expression Network Analysis

A co-expression network was constructed to evaluate relationships between DE mRNAs and DElncRNAs. The Pearson's correlation coefficient (PCC) between coding and non-coding genes was calculated. The mRNA–lncRNA pairs were selected if PCC ≥ 0.990. The network of lncRNA–mRNA interactions was visualized using the Cytoscape software (http://www.cytoscape.org/).

### Experimental Validation of Selected Co-expressed mRNA and lncRNA

The expression of selected lncRNA and mRNA was validated by qRT-PCR. Total RNA was reverse transcribed into cDNA using the PrimeScript RT Reagent Kit (Perfect Real Time; TaKaRa, Osaka, Japan) following kit instructions. cDNA was then subjected to qRT-PCR analysis on a QuantStudio 5 Real-Time PCR System using qPCR SYBR Green master mix (CloudSeq). The data were then normalized to the β-actin expression level. The relative expression ratios of selected co-expressed mRNA and lncRNA were calculated using the ΔΔCt method. The experiment was repeated three times. qRT-PCR primers of selected co-expressed mRNA and lncRNA are shown in Table 2.

**Table 2:** Primers for mRNA and lncRNAs.

**Genes**	**Type of primers**	**Sequences (5′-3′)**
**mRNA**		
ENSG00000099795	1-Forward	ACAGCTTCCCCAACTTCCTG
	1-Reverse	AACTCTGCCGCCTTCTTCTC
**lncRNA**		
ENST00000564683	1-Forward	GTCTTGAACTCCTGGGCTCA
	1-Reverse	GTGCCTCCGTTTTCCTCATC
ENST00000570408	1-Forward	AGGGAAGCAGAAACGAGACA
	1-Reverse	AGTTAATTCCGTGGGGCTCT
ENST00000628340	1-Forward	GCCAAACCCATAACAGTGCT
	1-Reverse	CCTCTCCTTCCTCAACGTCA

### Statistical Analysis

Graphs were plotted and analyses were performed by GraphPad Prism 8 software (San Diego, CA, USA). The statistical difference in relative expression ratios of the selected co-expressed mRNA and lncRNA between multiple groups was evaluated by one-way ANOVA followed by Tukey's multiple comparison test. The level of significance was set at *P* < 0.05.

## Results

### Demographic and Laboratory Test Data

The demographic and laboratory test data of 6 PWPs and 6 age- and sex-matched HCs are shown in Table 3. The PWPs were 52–68 years of age at early-stage (Hoehn and Yahr score 1.5–2.5) of PD for more than 2 years(range of 2–9 years). All laboratory tests of PWPs and HCs were within normal range.

**Table 3:** Clinical characteristics and blood biochemical features in PWPs and HCs.

	**PD-01**	**PD-02**	**PD-03**	**PD-04**	**PD-05**	**PD-06**	**HC-01**	**HC-02**	**HC-03**	**HC-04**	**HC-05**	**HC-06**
Sex (male/female)	Fmale	Male	Fmale	Fmale	Male	Fmale	Fmale	Male	Fmale	Male	Fmale	Fmale
Age (y)	67	55	68	62	52	66	65	52	67	55	62	64
Duration (y)	8	8	9	7	9	2	–	–	–	–	–	–
H and Y (score)	2.5	2	2.5	2	1.5	2	–	–	–	–	–	–
UPDRS III-off (score)	48	31	53	28	25	18	–	–	–	–	–	–
SBP (mmHg)	91	155	117	125	117	127	–	–	–	–	–	–
DBP (mmHg)	61	91	79	70	73	57	–	–	–	–	–	–
RBC (10^12^/L)	4.7	4.92	4.39	4.48	4.74	4.32	–	–	–	–	–	–
Hb (g/L)	140	153	127	135	154	130	–	–	–	–	–	–
WBC (10^9^/L)	6.85	6.45	5.67	7.44	4.6	7.68	–	–	–	–	–	–
NC (104^9^/L)	55.6	4.34	3.16	5.01	2.54	4.66	–	–	–	–	–	–
NR (%)	3.8	67.2	55.7	67.5	55.3	60.6	–	–	–	–	–	–
Plt (10^9^/L)	191	231	240	242	226	223	–	–	–	–	–	–
Na (mmol/L)	145	140	141	141	143	145	–	–	–	–	–	–
K (mmol/L)	4.2	3.8	3.7	4.1	4	4.1	–	–	–	–	–	–
Ca (mmol/L)	2.36	2.54	2.47	2.24	2.32	2.35	–	–	–	–	–	–
ALT (U/L)	12	20	26	15	24	9	–	–	–	–	–	–
AST (U/L)	18	16	19	18	22	17	–	–	–	–	–	–
TBIL (umol/L)	15.1	19.8	15.9	11.6	9.5	14.5	–	–	–	–	–	–
GHb (%)	6.6	7.1	6.6	5.9	5.4	5.8	–	–	–	–	–	–
Cre (umol/L)	64.8	62.2	61	60.1	80.3	72.6	–	–	–	–	–	–
BUN (mmol/L)	5.7	4.3	6.1	4.5	4.1	5.1	–	–	–	–	–	–

*PWPs, patients with PD; HCs, Healthy controls; H and Y, HoehnandYahr; UPDRS, Unified Parkinson Disease Rating Scale; SBP, systolic blood pressure; DBP, diastolic blood pressure; RBC, red blood cell count; Hb, hemoglobin; WBC, white blood cell count; NC, neutrophil count; NR, neutrophil ratio; Plt, platelet; Na, sodium ion; K, potassium ion; Ca, calcium ion; ALT, alanine transaminase; AST, aspartate aminotransferase; TBIL, total bilirubin; GHb, glycated hemoglobin; Cre, creatinine; BUN, blood urea nitrogen*.

The clinical and neuropsychological assessments of pre-and post-rehab PWPs are shown in Table 4. Post-rehab PWPs showed better and improved motor function (velocity and mean time of 10MWT, FTSST, 6MWT, TUG, the score of Mini-BESTest), cognition (the score of MMSE, MoCA), quality of life (the score of PDQ-39), and neuropsychological function (the score of HAMA, HAMD) compared with the pre-rehab PWPs.

**Table 4:** The results of motor, cognitive, quality of life, and neuropsychological assessment in the pre- and post-rehabilitation PWPs.

**Case No**.	**PD-01**		**PD-02**		**PD-03**		**PD-04**		**PD-05**		**PD-06**	
	**Pre-**	**Post-**	**Pre-**	**Post-**	**Pre-**	**Post-**	**Pre-**	**Post-**	**Pre-**	**Post-**	**Pre-**	**Post-**
**Motor functional assessment**												
10MV-V_CGS_ (m/s)	1.14	1.15	0.97	0.97	1.05	1.19	1.01	1.47	1.4	1.41	1.23	1.34
10MV-MT_CGS_ (s)	8.76	8.69	10.26	10.25	9.51	8.37	9.89	6.76	7.13	7.04	8.08	7.42
10MV-V_MGS_ (m/s)	1.82	1.87	1.86	1.43	1.61	1.68	1.24	1.91	2.04	2.42	1.54	1.95
10MV-MT_MGS_ (s)	5.46	5.34	5.35	6.96	6.18	5.94	8.02	5.22	4.88	4.12	6.46	5.1
FTSST (s)	9.37	10.03	12.1	9.91	9.09	10.99	10.61	8.9	6.92	5.49	8.72	7.87
6MWT (m)	522	527	445	520	466	446	509	536	559	591	464	498
TUG (s)	8.15	7.34	10.15	8.43	9.58	10.08	8.48	7.36	8.85	6.57	8.14	7.16
Mini-BESTest (score)	22	25	25	25	22	25	22	25	23	27	25	26
**Cognitive assessment**												
MMSE (score)	26	27	27	30	25	27	25	30	24	30	29	30
MoCA (score)	27	30	27	28	22	25	21	27	23	29	29	30
**Quality of life assessment**												
PDQ-39 (score)	39.74	23.71	23.07	14.1	20.51	18.58	14.1	13.46	21.15	14.74	17.3	12.17
**Neuropsychological assessment**												
HAMA (score)	15	4	4	4	5	9	10	9	6	6	12	8
HAMD (score)	12	4	7	4	2	8	5	3	6	1	8	4

*Pre-, pre- rehabilitation; Post-, post-rehabilitation; 10MWT-VCGS, the velocity of 10-m walk test in comfortable gait speed; 10 MWT-MTCGS, the mean time of 10-m walk test in comfortable gait speed; 10MWT -VMGS, the velocity of 10-m walk test in maximum gait speed; 10MWT -MTMGS, the mean time of 10-m walk test in maximum gait speed; FTSST, the Five Times Sit-to-Stand Test; 6MWT, the six-minute walk test; TUG, the time up and go; Mini-BESTest, Mini Balance Evaluation System Test; HAMA, Hamilton Anxiety Scale; HAMD, Hamilton depression scale; MMSE, Mini-mental State Examination; MoCA, Montreal Cognitive Assessment; PDQ-39, The Parkinson's Disease Questionnaire*.

### Differential Expression of Exosomal mRNAs and lncRNAs

**Figure 2:**
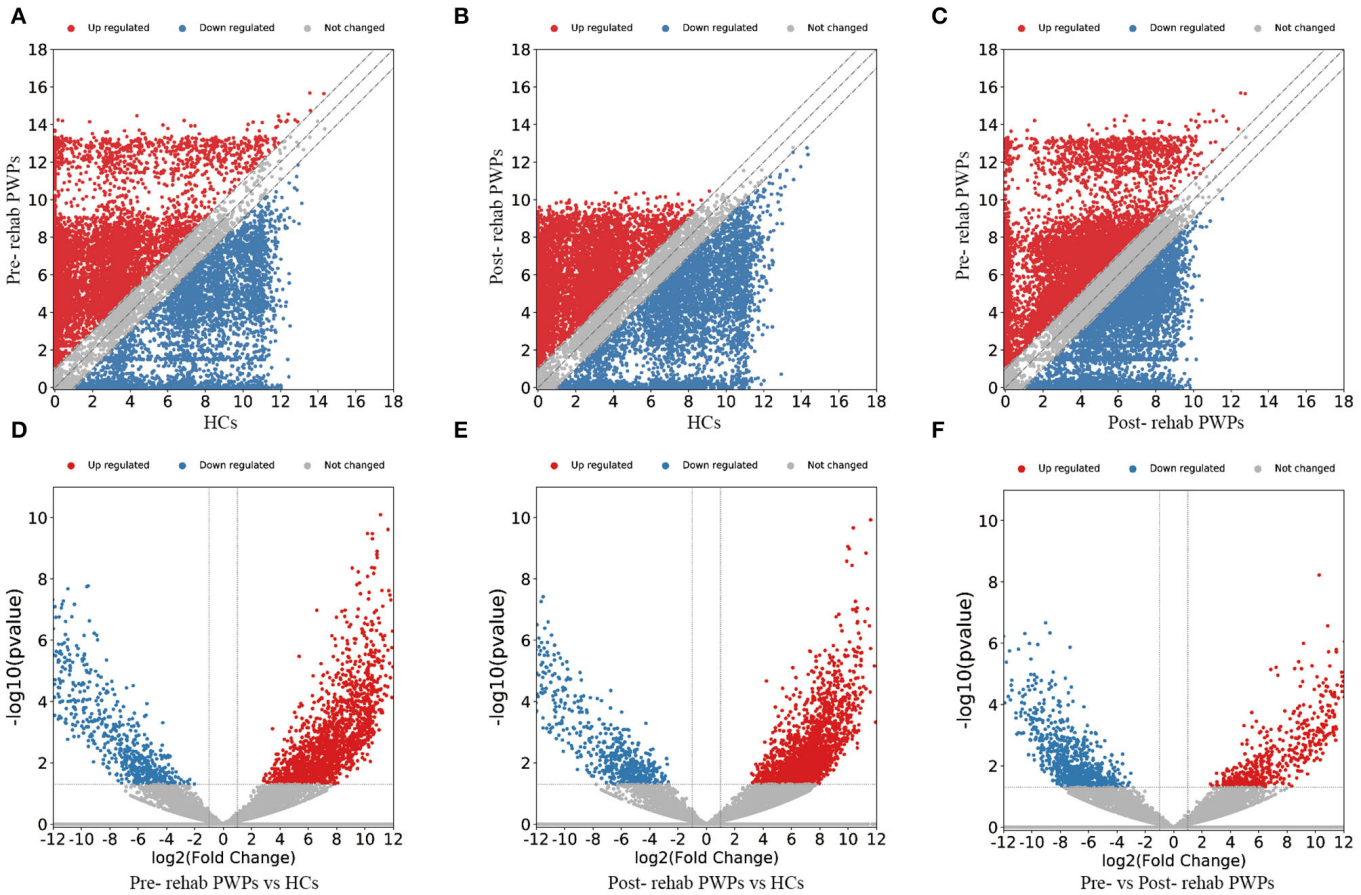
Changes in the mRNA expression profiles of the HCs, pre- and post-rehab PWPs. The scatter plot **(A)** and volcanic map **(D)** reveal that a significant difference existed in the distribution of mRNAs between pre-rehab PWPs and the HCs. The scatter plot **(B)** and volcanic map **(E)** reveal that a significant difference existed in the distribution of mRNAs between Post-rehab PWPs and the HCs. The scatter plot **(C)** and volcanic map **(F)** reveal that a significant difference existed in the distribution of mRNAs between pre- and post-rehab PWPs. Red represents upregulation, while green represents downregulation. Pre-rehab, pre-rehabilitation; Post-rehab, post-rehabilitation; PWPs, patients with Parkinson's disease.

**Figure 3:**
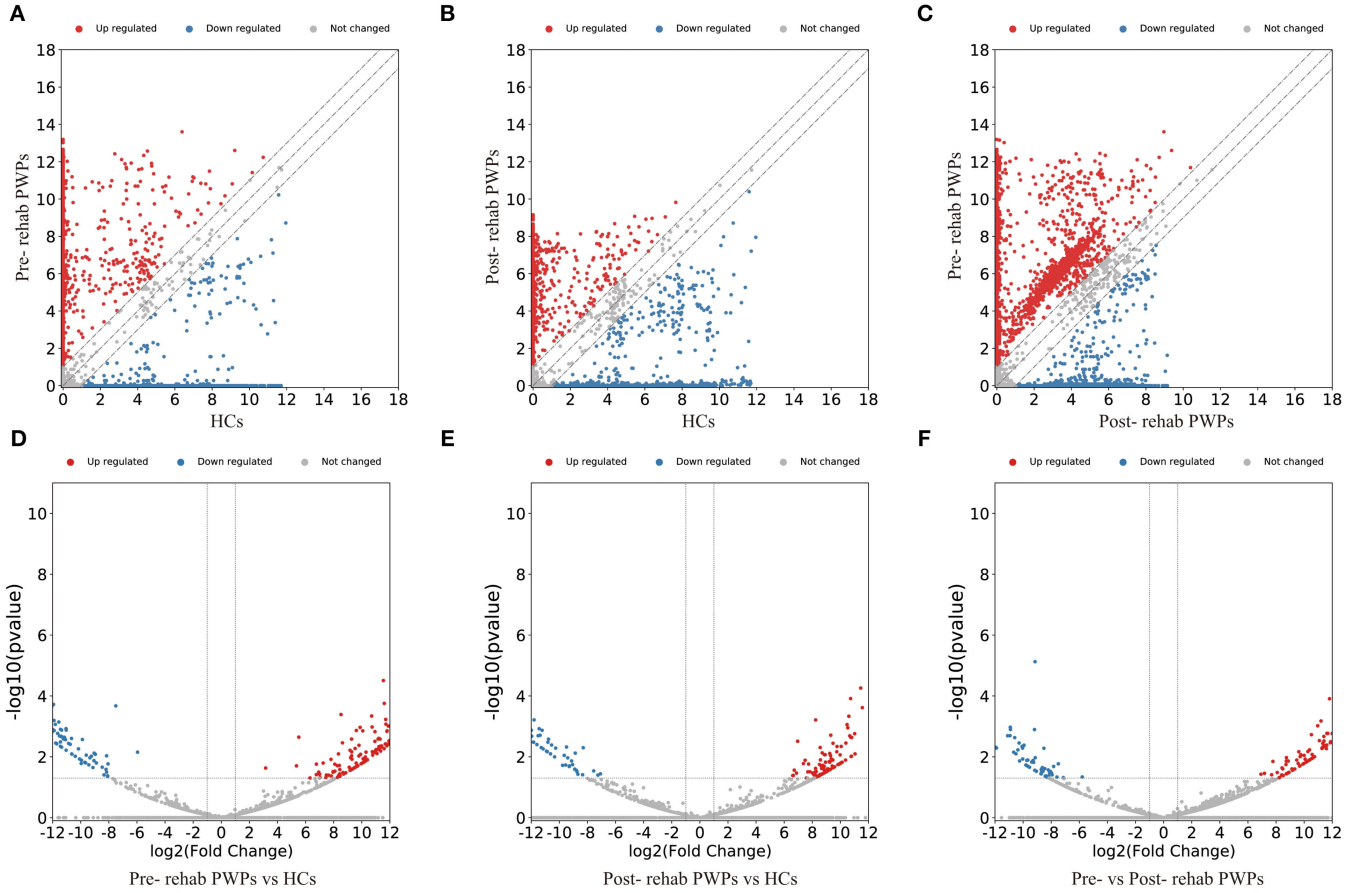
Changes in the lncRNA expression profiles of the HCs, pre- and post-rehab PWPs. The scatter plot **(A)** and volcanic map **(D)** reveal that a significant difference existed in the distribution of lncRNAs between pre-rehab PWPs and HCs. The scatter plot **(B)** and volcanic map **(E)** reveal that a significant difference existed in the distribution of lncRNAs between post-rehab PWPs and the HCs. The scatter plot **(C)** and volcanic map **(F)** reveal that a significant difference existed in the distribution of lncRNAs between Pre- and Post-rehab PWPs. Red represents upregulation, while green represents downregulation. Pre-rehab, pre-rehabilitation; Post-rehab, post-rehabilitation; PWPs, patients with Parkinson's disease.

The DEmRNAs and DElncRNAs are visually displayed in the form of scatter plots and volcano maps. Fold change ≥2.0 and *p* ≤ 0.05 were used as the thresholds for significantly differential expression. Compared with the HCs, there were 3,038 (including 2337 upregulated and 701 downregulated mRNAs) and 3,119 (including 2,490 upregulated and 629 downregulated mRNAs) DEmRNAs in the blood exosomes of pre-rehab (Figures 2A,D) and post-rehab PWPs (Figures 2B,E), respectively. Compared with pre-rehab PWPs, mRNA expression levels of post-rehab PWPs (Figures 2C,F) revealed 1,662 DEmRNAs, of which 606 and 1,056 were up and downregulated, respectively. In addition, lncRNA expression profiles revealed that, compared with the HCs, a total of 1,723 (including 1,278 upregulated and 445 downregulated lncRNAs) and 1,931 (including 1,561 upregulated and 370 downregulated lncRNAs) DElncRNAs were identified in the blood exosomes of pre- rehab (Figures 3A,D) and post-rehab (Figures 3B,E) PWPs, respectively. Compared with pre-rehab PWPs, lncRNA expression levels of post-rehab PWPs (Figures 3C,F) revealed 1,729 DElncRNAs, of which 593 and 1,136 were up and downregulated, respectively.

### GO Analysis of DEmRNAs

**Figure 4:**
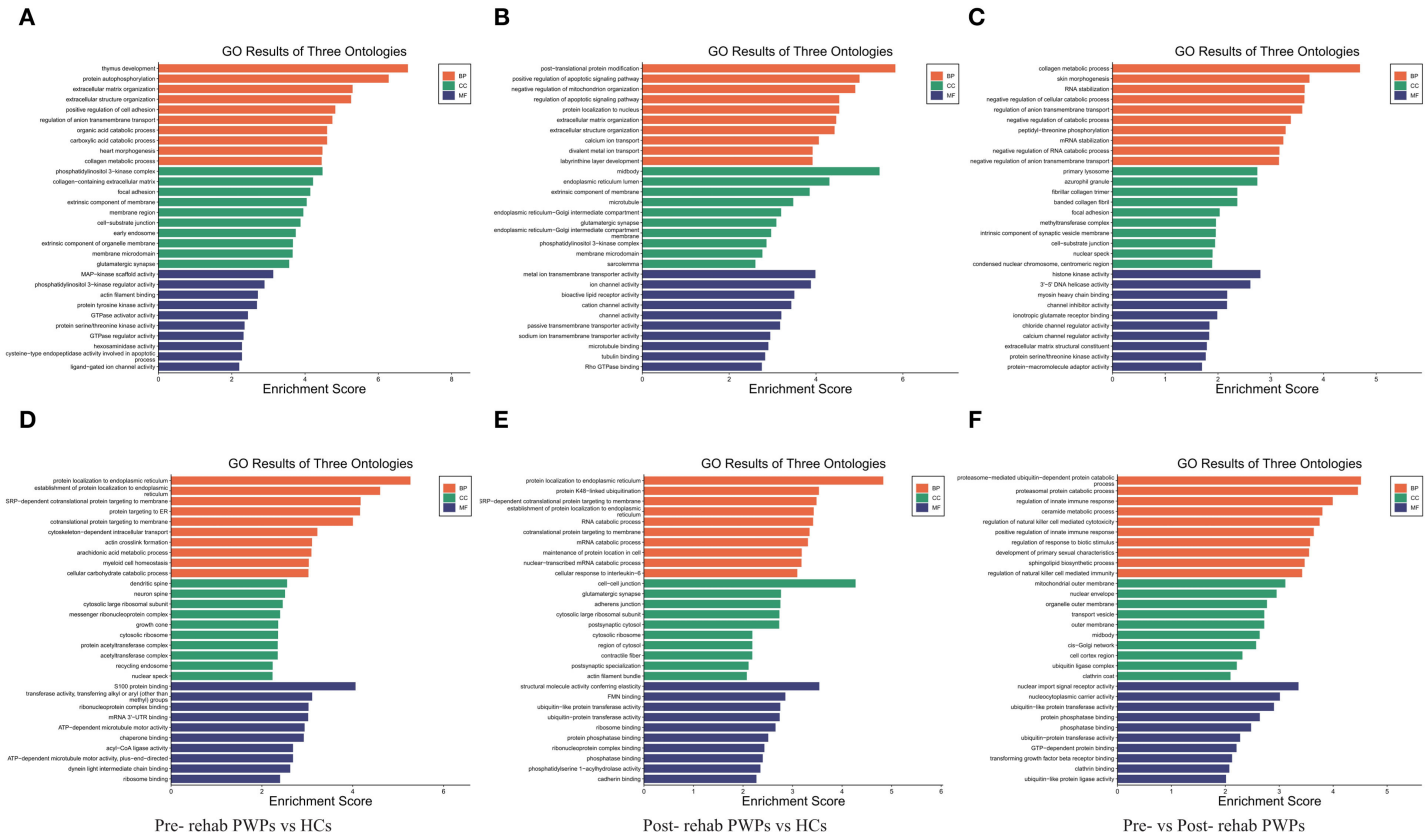
GO analysis of differentially expressed genes. GO analysis of upregulated **(A)** and downregulated **(D)** mRNAs between Pre-rehab PWPs and HCs. GO analysis of upregulated **(B)** and downregulated **(E)** mRNAs between Post-rehab PWPs and HCs. GO analysis of upregulated **(C)** and downregulated **(F)** mRNAs between Pre- and Post-rehab PWPs. Pre-rehab, pre- rehabilitation; Post-rehab, post-rehabilitation; PWPs, patients with Parkinson's disease; GO, gene ontology; BP, biological process, MF, molecular function, CC, cellular component.

Gene ontology enrichment analysis was performed to investigate the role of the DEmRNAs in the process of rehabilitation (Figure 4). GO analysis between HCs and pre-rehab PWPs revealed that the upregulated DEmRNAs were significantly enriched in biological processes related to thymus development, protein autophosphorylation, and extracellular matrix organization (Figure 4A), while the downregulated DEmRNAs were enriched in protein localization to the endoplasmic reticulum, establishment of protein localization to the endoplasmic reticulum, SRP–dependent cotranslational protein targeting the membrane (Figure 4D).

Gene ontology analysis between HCs and post-rehab PWPs revealed that the upregulated DEmRNAs were significantly enriched in post–translational protein modification, positive regulation of apoptotic signaling pathway, and negative regulation of mitochondrion organization (Figure 4B). The downregulated mRNAs were enriched in protein localization to the endoplasmic reticulum, protein K48–linked ubiquitination, SRP-dependent cotranslational protein targeting the membrane (Figure 4E).

Gene ontology analysis between pre-and post-rehab PWPs revealed that the upregulated DEmRNAs were significantly enriched in collagen metabolic process, skin morphogenesis, and RNA stabilization (Figure 4C). These downregulated DEmRNAs are involved in proteasome-mediated ubiquitin-dependent protein catabolic process, proteasomal protein catabolic process, and the regulation of innate immune response (Figure 4F).

### Pathway Analysis of DEmRNAs

**Figure 5:**
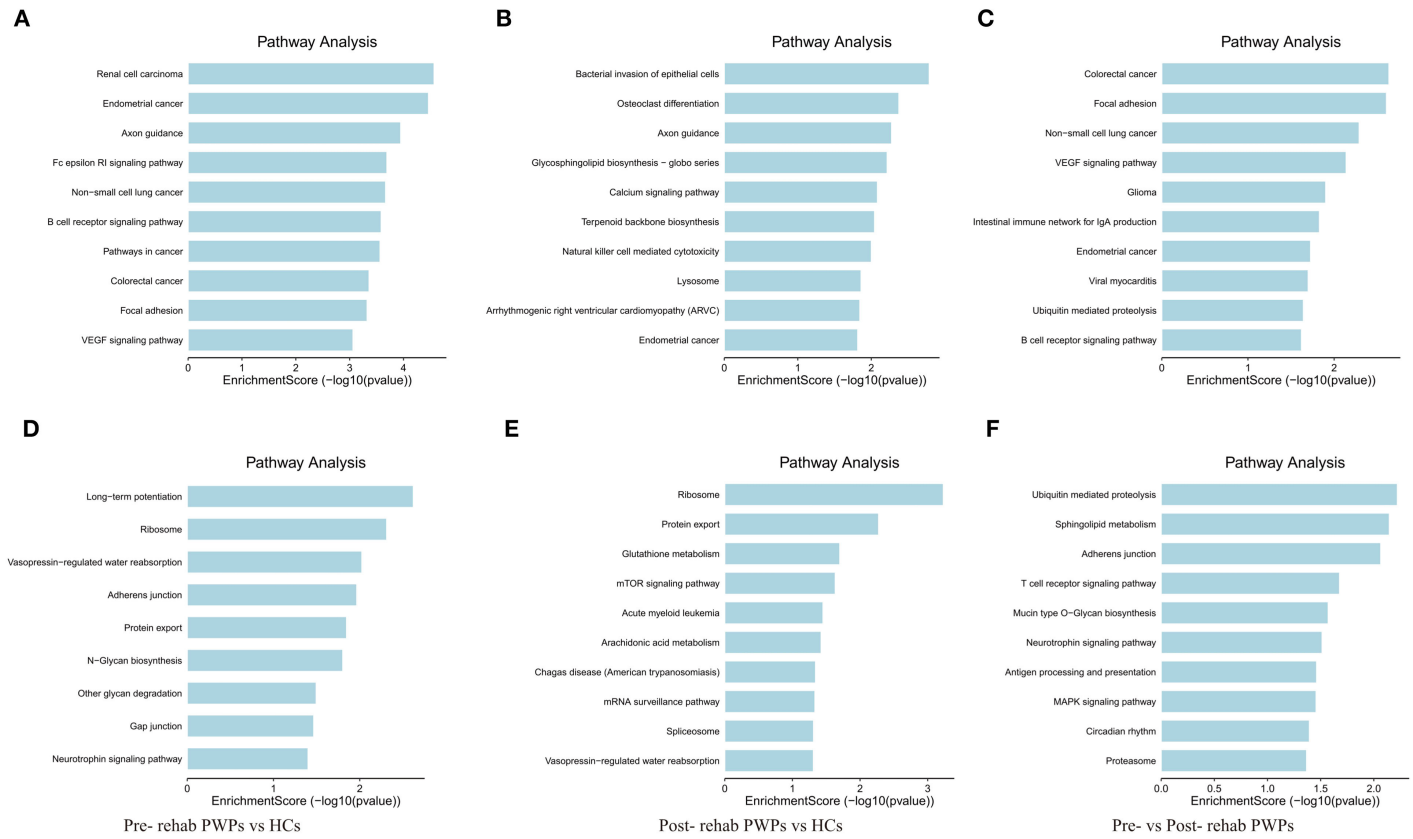
KEGG pathway analysis of differentially expressed genes. KEGG pathway analysis of upregulated **(A)** and downregulated **(D)** mRNAs between pre-rehab PWPs and HCs. KEGG pathway analysis of upregulated **(B)** and downregulated **(E)** mRNAs between pre-rehab PWPs and HCs. KEGG pathway analysis of upregulated **(C)** and downregulated **(F)** mRNAs between pre- and post-rehab PWPs. Pre-rehab, pre-rehabilitation; post-rehab, post-rehabilitation; PWPs, patients with Parkinson's disease; KEGG, Kyoto Encyclopedia of Genes and Genomes.

KEGG pathway analysis identified the pathways influenced by exosomal DEmRNAs of HCs, pre- and post-rehab PWPs. The pathways of upregulated DEmRNAs between HCs and pre-rehab PWPs are involved in “axon guidance,” “Fc epsilon RI signaling pathway,” “B cell receptor signaling pathway” (Figure 5A), while the pathways of downregulated DEmRNAs are involved in “long–term potentiation,” “ribosome,” and “neurotrophin signaling pathway” (Figure 5D).

Compared to the HCs, the pathways associated with upregulated DEmRNAs in post-rehab PWPs are involved in “lysosome,” “natural killer cell mediated cytotoxicity,” “calcium signaling pathway” (Figure 5B), while the pathways associated with downregulated DEmRNAs are involved in the “mRNA surveillance pathway,” “mTOR signaling pathway,” and “protein export” (Figure 5E). Then compared to the pre-rehab PWPs, the pathways associated with upregulated DEmRNAs of the post-rehab group are involved in the “B cell receptor signaling pathway,” “ubiquitin-mediated proteolysis,” “VEGF signaling pathway” (Figure 5C), while the pathways associated with downregulated DEmRNAs are involved in the “MAPK signaling pathway,” “neurotrophin signaling pathway,” and “proteasome” (Figure 5F).

### Co-expression of DElncRNAs and DEmRNAs

**Figure 6:**
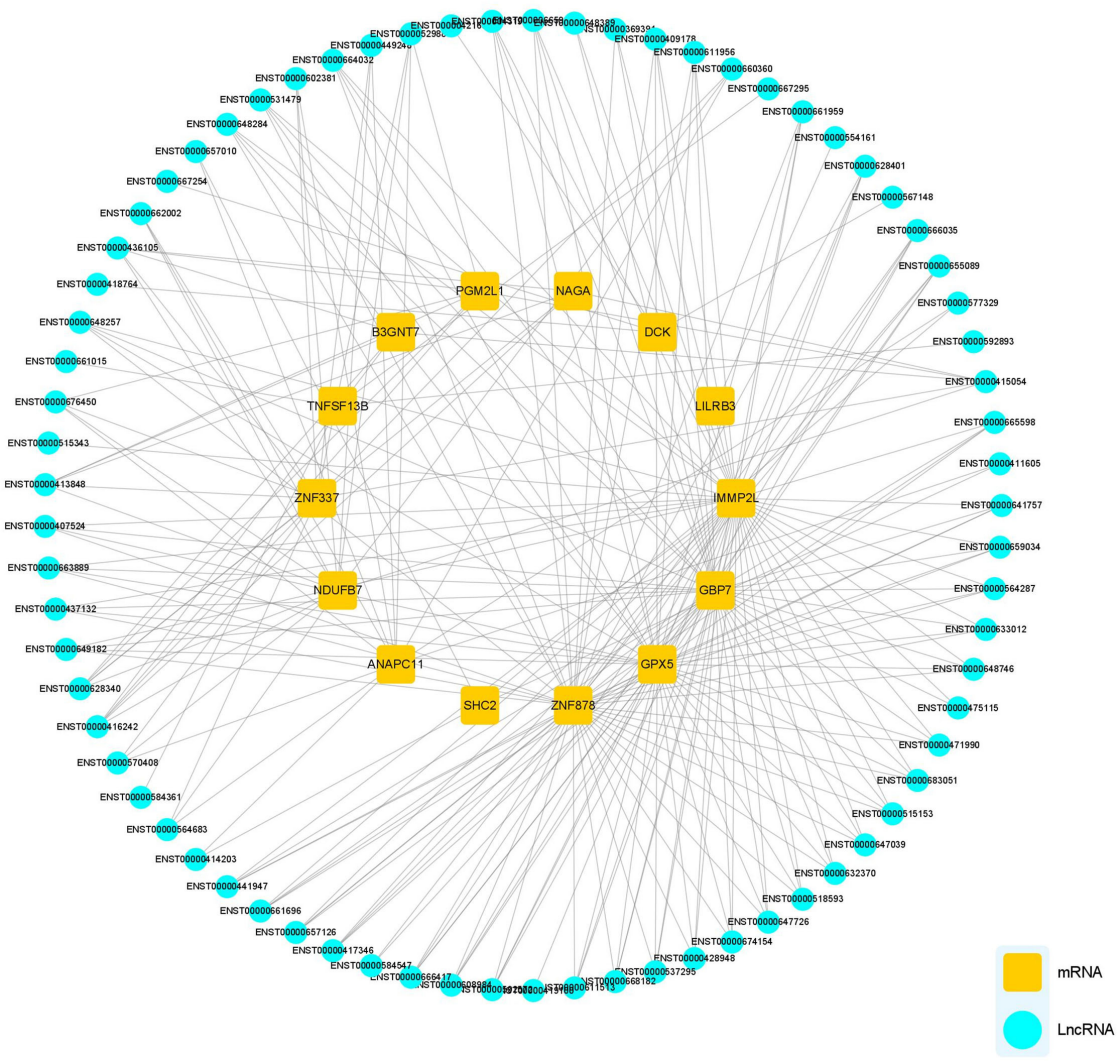
lncRNA-mRNA Co-expression network analysis. lncRNA-mRNA co-expression networks contained 87 nodes, including 73 lncRNAs and 14 mRNA DEmRNAs which were positively related. lncRNAs, long noncoding RNAs; mRNA, messenger RNA; DElncRNAs, differentially expressed lncRNAs; DEmRNAs, differentially expressed mRNAs.

The potential functions of mRNA can be inferred from lncRNA–mRNA co-expression networks. We screened all DEmRNA and DElncRNA, and 14 DEmRNAs and 73 DElncRNAs of interest were expressed simultaneously in HCs, pre-and post-rehab PWPs. The lncRNA–mRNA co-expression revealed that the interaction network comprised 87 nodes, including 14 DEmRNAs and 73 targeted DElncRNAs. These nodes formed 243 positive network pairs (Figure 6). The network displays that a single mRNA may be correlated with several lncRNAs and vice versa.

### Experimental Validation of Selected lncRNAs and mRNAs

**Figure 7:**
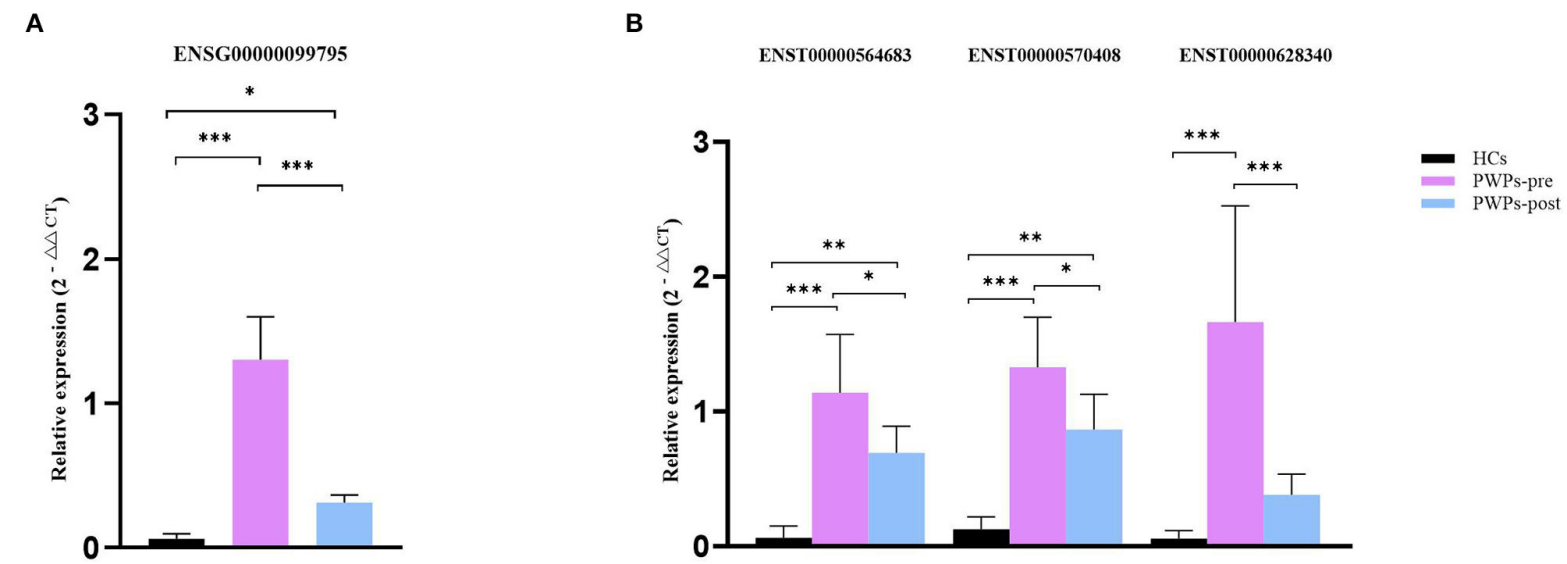
qRT-PCR validation of differentially expressed mRNA and lncRNAs. qRT-PCR verification of the expression profiles of NDUFB7 mRNA **(A)** and 3 related lncRNAs **(B)** in HCs, pre- and post-rehab PWPs. ^*^P < 0.05, ^**^P < 0.01, ^***^P < 0.001. Pre-rehab, pre-rehabilitation; post-rehab, post-rehabilitation; PWPs, patients with Parkinson's disease.

For validation purposes, we selected one DEmRNA (ENSG00000099795, NDUFB7) of interested and three related DElncRNAs (ENST00000564683, ENST00000570408, and ENST00000628340) from the co-expression pairs of DEmRNAs and DElncRNAs. The housekeeping gene β-actin was used as an internal control for normalization. NDUFB7 encodes a subunit of reduced nicotinamide adenine dinucleotide (NADH) dehydrogenase-ubiquinone oxidoreductase (complex I), which is a marker related to mitochondrial respiration. The qRT-PCR results were consistent with our sequencing results for the variation of DEmRNA (Figure 7A) and DElncRNAs (Figure 7B). According to qRT-PCR results, the expression of selected exosomal DEmRNA was significantly upregulated in pre-rehab PWPs (*P* < 0.0001) and post-rehab PWPs (*P* = 0.0475) compared with HCs, and significantly downregulated in post-rehab PWPs compared with pre-rehab PWPs (*P* < 0.0001).

Meanwhile, compared with HCs, all of the selected exosomal DElncRNAs were expressed at a significantly higher level (all *P* ≤ 0.0001) in pre-rehab PWPs and two of the selected exosomal DElncRNAs, (ENST00000564683, *P* = 0.0025; ENST00000570408, *P* = 0.0005) expressed at a significantly higher level in post-rehab PWPs. All of the selected exosomal DElncRNAs were expressed at a significantly lower level in the post-rehab PWPs than in pre-rehab PWPs (*P* < 0.05).

## Discussion

This pilot study included 6 early-stage PWPs and 6 age- and sex-matched HCs. The PWPs responded well to rehabilitation treatment based on comprehensive clinical assessment, including motor behavior, cognition, quality of life, anxiety, and depression. Rehabilitation seems to be a promising non-pharmacological therapy for PD (Goodwin et al., 2008). Some studies have previously reported that rehabilitation measures could lead to significant improvement in motor function, including walking ability, posture, and balance control for PWPs (Ashburn et al., 2007; Chen K. K. et al., 2021). However, the underlying mechanisms for improvement of PWPs after rehabilitation is not clear.

High-quality biomarkers are important for PD diagnosis, monitoring, and treatment response evaluation (Cao et al., 2017). Exosomes are extracellular vesicles (EVs) secreted by multiple cells and contain cargos including protein, lncRNA, microRNA (miRNA), and mRNA. Exosomes will transfer to the central nervous system (CNS) and release to exert pleiotropic effects (Deng et al., 2020). Exosomes can take part in many biological processes and contribute to intracellular communication, which makes them important in neurodegenerative diseases (Kalluri and Lebleu, 2020). Previous analysis of exosomes isolated from the blood or CSF of PWPs suggested that exosomes may be valid biomarkers of PD (Vella and Hill, 2016). As a result, we quantitatively analyzed the exosomal mRNA and lncRNA expressions in peripheral blood of PWPs and HCs using NGS and real-time quantitative PCR.

Gene ontology analysis revealed that the DEmRNAs were significantly associated with the biological processes such as regulation of mitochondrion organization, regulation of apoptotic signaling pathway, and calcium ion transport. The significantly enriched CC term included mitochondrial outer membrane, primary lysosome, and dendritic spine. The significantly enriched MF term comprised cysteine–type endopeptidase activity involved in the apoptotic process, ubiquitin–like protein ligase activity, and ribosome binding. The functional annotations demonstrated that the aberrantly expressed genes participate in mitochondrial dysfunction-related pathologic processes in PD, such as oxidative stress response, ubiquitin system, apoptosis, and calcium signaling (Zhang et al., 2010). KEGG pathway analysis revealed that DEmRNAs were involved in several biological pathways, of which protein processing in the calcium signaling pathway and the mTOR signaling pathway is related to mitochondrial metabolism in PD (Zhu et al., 2019). Thus, it is reasonable to consider that mitochondrial-related exosomal mRNA and lncRNA are implicated in the pathogenesis and rehabilitation of PD.

One DEmRNA of interest (NDUFB7) and three related DElncRNAs were selected to explore the potential role of the mRNA and lncRNAs in this process. NDUFB7 mRNA, encoding a subunit of reduced NADH dehydrogenase-ubiquinone oxidoreductase (complex I), was positively related to three DElncRNAs. Previous reports were consistent in describing a selective deficiency of respiratory chain complex I in the substantia nigra (SN) of PWPs (Janetzky et al., 1994). Adenosine triphosphate is generated through the mitochondrial electron transport chain (ETC) and the oxidative-phosphorylation system (Perier and Vila, 2012). In the process of oxidative phosphorylation, complex I works as the entry point for electrons being transmitted from mitochondrial matrix to the ETC by catalyzing the electron transfer from NADH into the ETC subunits (Subramaniam and Chesselet, 2013). A reduction in electron transfer capacity was observed in patients with PD indicating that oxidative damage to mitochondria may result in complex I impairment (Keeney et al., 2006). MPTP (1-Methyl-4-phenyl-1,2,5,6-tetrahydropyridine) induced inhibition of mitochondrial CI, and subsequent DA neuronal loss has been reproduced in mammalian models such as primates and mice. Post-mortem studies have suggested a complex I deficiency in the SN of patients with PD, reflecting the mitochondrial injury of the neuronal cells (Subrahmanian and Lavoie, 2021). So, we assumed that mitochondrial pathway of NDUFB7 mRNA may be involved in the rehabilitation of PD. Some lncRNAs also play an important role in PD by promoting or inhibiting translation (Chen Y. et al., 2021). In our study, the selected lncRNAs positively regulated the selected mRNA, suggesting that the lncRNAs also play important roles in expression and regulation of the genes implicated in PD rehabilitation. However, exosomal lncRNAs have not been investigated in depth and little is known about the role of exosomal lncRNAs in the pathogenesis of PD.

The qRT-PCR validation for the DEmRNA of interest and related DElncRNAs revealed upregulated expression level of NDUFB7 in pre-rehab PWPs compared to post-rehab PWPs. The release of damage-related molecules of mitochondria is a potential mechanism connecting mitochondrial dysfunction with systemic inflammation in the setting of various diseases, such as PD (Currim et al., 2021). A previous study indicated that mildly damaged mitochondria were primed by serine/threonine-protein kinase PINK1 and Parkin and generated mitochondrial-derived vesicles (MDVs; Mclelland et al., 2014). MDVs containing mitochondrial components are secret out of the endolysosomal system and are released into the extracellular compartment as exosomes (Matheoud et al., 2016). Therefore, we suppose that the benefits of rehabilitation for PWPs may involve the restoration of mitochondrial function, and NDUFB7 is a potential biomarker for the evaluation of the effect of PD rehabilitation.

Several limitations may affect the interpretation of our findings. The insufficient sample size in our study may undermine our conclusion. More convincing evidence is required to validate the conclusion with a larger sample size. Our results suggested downregulated expression of NDUFB7 mRNA in favor of the post-rehab PWPs, but the difference between pre-rehab and post-rehab PWPs was not statistically significant. Better baseline motor function of PWPs may be partially responsible for this phenomenon. The functions of DElncRNAs were only predicted through the co-expression analyses. The precise underlying mechanisms have not been clarified yet. Additional studies are required to investigate the exact roles of exosomal lncRNAs in PD. Future research will clarify how NDUFB7 may contribute to PD pathogenesis and how rehabilitation treatment down-regulates NDUFB7 expression at cellular and animal levels.

## Conclusion

NDUFB7 mRNA and three related lncRNAs are potentially associated with the rehabilitation of PD by restoring mitochondrial function. These findings provide valuable insight into the potential biomarkers pertinent for evaluating the treatment strategies for PD.

## Data Availability Statement

The data presented in the study can be found in online repositories. The names of the repository/repositories and accession number(s) can be found below: https://ngdc.cncb.ac.cn/gsa-human/, HRA002383.

## Ethics Statement

The studies involving human participants were reviewed and approved by the Ethics Committee of Beijing Rehabilitation Hospital of Capital Medical University (2020bkky010). The patients/participants provided their written informed consent to participate in this study.

## Author Contributions

BF conceived and designed the study. YL and YW performed the experiments and statistical analysis. YL, ZJ, CL, XY, and KC collected patients' data. YW wrote the first draft of the manuscript. BF, AL, ZJ, and DM reviewed and edited the manuscript. All authors contributed to the manuscript revision and read and approved the submitted version.

## Funding

This study was supported by the Science and Technology Development Fund of Beijing Rehabilitation Hospital, Capital Medical University (2019-023 to YL, 2020-069 to BF, and 2020R-001 to YW). The funding body had no role in study design, statistical analysis, and manuscript preparation.

## Conflict of Interest

The authors declare that the research was conducted in the absence of any commercial or financial relationships that could be construed as a potential conflict of interest.

## Publisher's Note

All claims expressed in this article are solely those of the authors and do not necessarily represent those of their affiliated organizations, or those of the publisher, the editors and the reviewers. Any product that may be evaluated in this article, or claim that may be made by its manufacturer, is not guaranteed or endorsed by the publisher.

## Supplementary Material

Supplementary Figure 1 Identification of exosomes. Transmission electron microscopy. White arrow heads point to exosome **(A)**. Western blotting exosomes are positive for CD9 **(B)** and CD81 **(C)**.
