# Supplementary figures and images for: Association Between Mitochondrial Function and Rehabilitation of Parkinson's Disease: Revealed by Exosomal mRNA and lncRNA Expression Profiles
Source: Front Aging Neurosci. 2022 Jun 16;14:909622. doi: 10.3389/fnagi.2022.909622 (PMC9244703; doi:10.3389/fnagi.2022.909622)

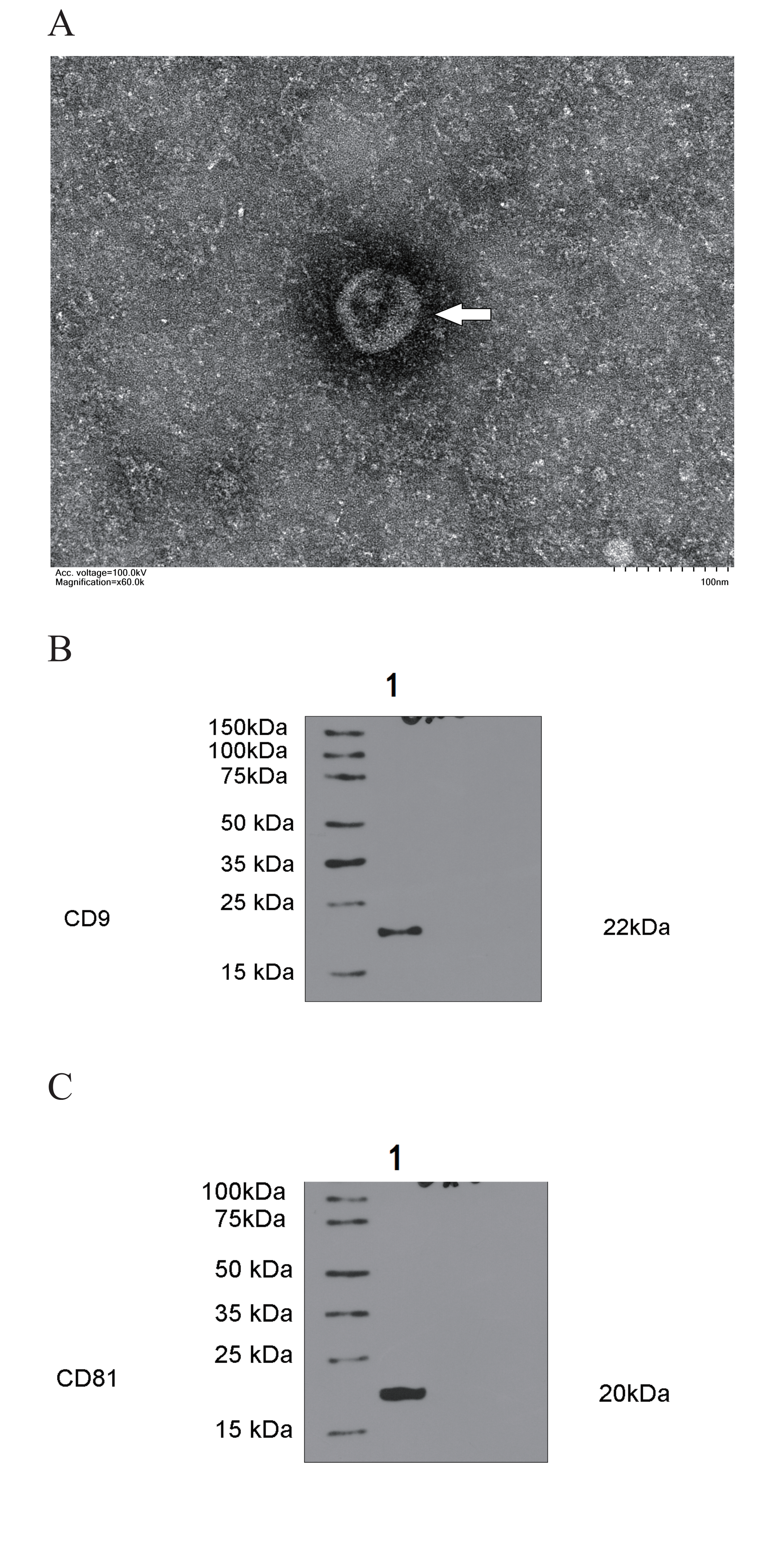

Supplement: Supplementary Figure 1 — Identification of exosomes. Transmission electron microscopy. White arrow heads point to exosome (A). Western blotting exosomes are positive for CD9 (B) and CD81 (C). [file Image_1.TIF]
