# Supplementary material for: Association Between Mitochondrial Function and Rehabilitation of Parkinson's Disease: Revealed by Exosomal mRNA and lncRNA Expression Profiles
Source: Front Aging Neurosci. 2022 Jun 16;14:909622. doi: 10.3389/fnagi.2022.909622 (PMC9244703; doi:10.3389/fnagi.2022.909622)
Supplement: Supplementary file 2 [file Table_1.DOCX]

**MATERIALS AND METHODS**

**Isolation and Identification of Exosomes**

The exoEasy Maxi Kit (Qiagen, Hilden, Germany) was used to isolate the exosomes. Particles in blood samples larger than 0.8 μm were excluded. Phosphate-buffered saline (1 ml) was added to the sample. The mixture was then added to the exoEasy column and centrifuged at 500 × g for 1 minute at 37°C, the bottom layer was discarded. Washing buffer (10 mL) was added to the column. The eluate was centrifuged at 500 ×g for 5 minutes and immediately stored at -80°C until later use.

The exosomes were identified using transmission electron microscopy (TEM) (Hitachi HT-7800, Tokyo, Japan) and western blot analysis. Specific exosomes were characterized by the presence of several exosomal markers, including the cluster of differentiation 9 (CD9) and the cluster of differentiation 81 (CD81). β-actin was used as the negative control.

**RESULTS**

**Characterization and Properties of Exosomes**

TEM images of exosomes showed exosomes as small cap-shaped membrane vesicles with a mean diameter ranging from 60 to 100 nm (Figure S1A). Western blot analysis revealed the presence of CD9 (Figure S1B) and CD81(Figure S1C), which were exosomal surface protein markers.
